# Holography with Photochromic Diarylethenes

**DOI:** 10.3390/ma12172810

**Published:** 2019-09-01

**Authors:** Luca Oggioni, Giorgio Pariani, Frédéric Zamkotsian, Chiara Bertarelli, Andrea Bianco

**Affiliations:** 1 INAF-Osservatorio Astronomico di Brera, via E. Bianchi 46, 23807 Merate (LC), Italy; 2 Politecnico di Milano, Dipartimento di Chimica, Materiali e Ingegneria Chimica ‘Giulio Natta’, P.zza L. da Vinci 32, 20133 Milano (MI), Italy; 3 Aix Marseille Universite, CNRS, CNES, LAM, Laboratoire d’Astrophysique de Marseille, 38 Rue Frédéric Joliot Curie, 13388 Marseille CEDEX 13, France

**Keywords:** holography, photochromism, diarylethenes, refractive index, CGH

## Abstract

Photochromic materials are attractive for the development of holograms for different reasons: they show a modulation of the complex refractive index, meaning they are suitable for both amplitude and phase holograms; they are self-developing materials, which do not require any chemical process after the light exposure to obtain the final hologram; the holograms are rewritable, making the system a convenient reconfigurable platform for these types of diffractive elements. In this paper, we will show the features of photochromic materials, in particular diarylethenes in terms of the modulation of a transparency and refractive index, which are mandatory for their use in holography. Moreover, we report on the strategies used to write binary and grayscale holograms and their achieved results. The outcomes are general, and they can be further applied to other classes of photochromic materials in order to optimize the system for achieving high efficiency and high fidelity holograms.

## 1. Introduction

The possibility of storing a 3D scene in a substrate has been a dream for a long time. Thanks to Gabor and his invention of holography in 1948 [1] and laser development in the following decades [2], such a dream has come true. Since then, holography has found many potential technological uses, while important developments for both theory and application have been achieved [3]. 

When considering the hologram manufacturing, issues related to photosensitive material are crucial. Indeed, an ideal material for hologram manufacturing should show [4]: a high spatial resolution, a large dynamic range, a good signal to noise ratio, high optical quality, and large sensitivity in a wide spectral range. Another attractive property that holographic materials may show is the ability to self-develop, namely, no chemical process is required after the pattern transfer to obtain the final usable hologram. Clearly, the choice of the photosensitive material depends on different factors, in particular, if the hologram is a phase or amplitude, and the technique used to transfer the pattern. In addition, strategies to obtain holograms that are reconfigurable and switchable are highly desired.

There are different approaches to achieve these kinds of diffractive devices and photochromic dyes surely are an interesting option. Nice features of such materials include their rewritability, which is intrinsic in the reversible transformation. Moreover, they can be used for making both amplitude and phase holograms [5]. Among the different classes of photochromic materials, T-type materials are interesting in the case of real-time holography because of their efficient thermal decoloration process [6]; whereas the P-type (thermally stable) holograms are much more interesting where re-addressable holograms are required. Diarylethenes are surely the most studied holograms for holographic optical memories, 3D displays, and holographic gratings [7,8,9,10,11,12] belonging to the P-type class, thanks to their well-known good overall photochromic properties [13] and the possibility of obtaining highly responsive films. In addition, the use of diarylethenes in combination with nanoparticles (in particular gold ones) could be of great interest in this field, since the optical properties and their switching can be tuned by acting both on the nanoparticles side (mainly size and dispersion, which affect the plasma frequency) and the photochromic unit side [14,15,16,17,18,19]. The performances of diarylethene based holograms are strongly related to the optimization of the photochromic substrate and to the writing procedure. 

Other strategies and materials are possible to use to obtain reconfigurable holograms: photorefractive materials and photosensitive liquid crystals are two interesting families. As for photorefractive materials, they show a refractive index modulation as the result of the photoconductive and the Pockels effects [20], which makes them suitable for phase holographic elements [21]. Fast reconfigurable holograms [22,23], 3D holographic displays [24,25], and holographic memories [26] can be obtained thanks to the rapid growth of refractive index modulation, a very peculiar characteristic of such systems. On the other hand, the hologram is not usually persistent, so this approach is not suitable for long lasting devices. Regarding holographic liquid crystals (LCs), there are different possibilities since the LCs can change their properties (orientation, phase separation, and refractive index modulation) through both optical stimuli and electric stimuli. Consequently, rewritable systems, ON-OFF grating, and polarized sensitive gratings are possible. In the case of light sensitive LCs, azobenzene photochromic moieties are often used [27,28] and rewritable holograms can be obtained by achieving major modulation of the refractive index. Similar systems were also considered for making holographic memories [29]. By using polymer-dispersed liquid crystal combined with holography (H-PDLC), it is possible to obtain switchable phase gratings and other optical elements thanks to the phase separation that induces the refractive index modulation and the application of the electric field [30,31].

In this paper, we report on the main features of photochromic diarylethenes in terms of relevant properties for phase and amplitude holograms. A hybrid computation tool is shown to help the optimization of the films, mainly focusing on the chemical structure of the diaryelethene; the different strategies for writing photochromic holograms are also discussed. Examples are reported in order to support the discussion. The results here reported can be easily generalized to other classes of photochromic materials and could inspire the development of new/optimized photochromic systems for high efficiency and high fidelity holographic optical elements.

## 2. Computer-Generated Holograms

Holograms digitally calculated are called Computer-Generated Holograms (CGHs). The ideal wavefront to be reconstructed is computed on the basis of the diffraction theory, starting from the wave field distribution of the object beam [32,33]. Such an approach is of great interest since it allows for recording holograms of virtually any object or scene without the existence of the physical object. They can also use optical elements and filters to manipulate light phase and intensity. In the same manner as traditional holograms, CGHs are classified as either phase or amplitude. 

In the scalar diffraction approach, if we neglect the reconstruction noise, depending on the hologram type and discretization levels, both types of hologram are able to reconstruct the desired object with the main difference being in the diffraction efficiency [32], as will be discussed later on (see Section 2.2). When diffraction efficiency is not an issue, amplitude holograms may be preferred for their easier manufacture. CGHs, thanks to their ability to generate custom wavefronts, are finding applications in beam shaping, particles manipulation, interferometric optical testing, and anti-counterfeiting [34,35,36].

Concerning interferometry, great efforts have been done in the recent years to improve CGH capabilities beyond their first development by Wyant almost 50 years ago [37]. Nowadays, they are used as optical surface references, to cope with the production of complex and non-standard optical surfaces (aspherical and free form), which are made possible by new optical fabrication technologies [38]. They are applied to test different optics, even large aspherical mirrors for the new generation telescopes [39,40].

Two main type of holograms can be recorded in the holographic material [41]: the Fourier hologram, which exploits the inverse Fourier transformation of the image, and the Fresnel hologram, which encodes the interference pattern of the wave propagated to the object.

### 2.1. Fourier and Fresnel CGHs

A collimated beam passing through a lens undergoes a Fourier transformation. Thus, starting from the image to be reconstructed, it is possible to calculate the complex wavefront to be encoded in the CGH using Equation (1). Moreover, the inverse transformation, can be used to reconstruct the image from the CGH pattern [42]. Here, we report the mathematical operator of the direct ℱ and inverse ${ℱ}^{-1}$ Fourier Transformations:

(1) $$g\left(\mu ,\nu \right)=ℱ\left[f\right]\left(\mu ,\nu \right)=\iint_{R^{2}}^{\;}f\left(x,y\right)\;e^{-2\pi i\left(x\mu +y\nu \right)}dxdy$$

(2) $$f\left(x,y\right)={ℱ}^{-1}\left[g\right]\left(x,y\right)=\iint_{R^{2}}^{\;}g\left(\mu ,\nu \right)\;e^{2\pi i\left(x\mu +y\nu \right)}d\mu d\nu$$

where *x, y* and μ,ν are the coordinates of the image plane and Fourier space, respectively.

Fresnel holograms are directly calculated by propagating the wavefront to be reconstructed and exploiting the light propagation equations that are modeled by the Rayleigh-Sommerfeld diffraction theory [43]. If we consider to have the hologram plane in *z* = 0 and the object plane at *z*, we can write:

(3) $$E_{z}\left(x,y\right)=\iint^{\;}E_{0}\left(u,v\right)\frac{\;e^{ikr}}{r}dudv\;$$

The resulting complex wave *E_z_* is estimated by calculating the sum of the contributions of each pixel of the hologram anywhere on the screen located at a distance *z*. Each pixel is considered as a secondary spherical wave source weighted by the function *E*_0_(u,ν). These secondary waves are generated when the incident wave, characterized by its complex amplitude *E*_0_ and wavelength *λ*, reaches the hologram. The same strategy can be applied to calculate *E*_0_ by inverting Equation (3) and positioning the object plane at −*z*, in order to keep the same direction of propagation.

(4) $$E_{0}\left(u,\nu \right)=\iint^{\;}E_{-z}\left(x,y\right)\frac{\;e^{ikr}}{r}dxdy\;$$

The function $E_{0}\left(u,v\right)$ is the complex amplitude of the hologram, which must be approximated before the encoding.

### 2.2. Diffraction Efficiency

Once the complex electric field function at the hologram plane has been calculated, the next step is the encoding into the CGH. However, to transfer all the complex information, a material able to modulate both amplitude and phase of a wavefront is needed. Despite some successful attempts, multi-step processes and complex procedures are ultimately required for this approach [44]. The traditional approach is to code the complex wavefront in the form of a phase only or amplitude only map. The main difference between the two coding strategies is the hologram diffraction efficiency. 

**Figure 1:**
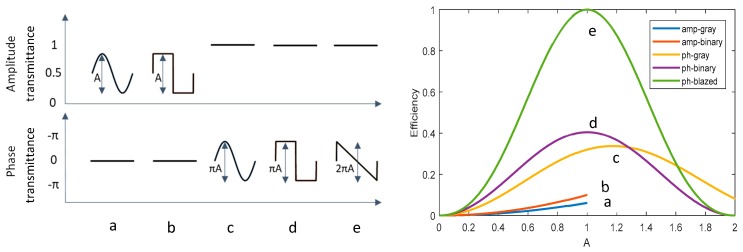
Modulation profiles for grayscaled (**a**), binary (**b**) amplitude holograms, grayscaled (**c**), binary (**d**) and blazed (**e**) phase holograms. Dependence of the first order diffraction efficiency on the modulation parameter A (right).

In order to give an estimation of the hologram efficiency, we make use of a model reported by Brown in 1969 [32], which considers monodimensional gratings with a periodical structure that is either binary or grayscaled. The wavelength of the incident light is assumed to be much smaller than the grating period, so the scalar diffraction approximation can be applied. Figure 1 reports the theoretical efficiency of gray-scaled (a) and binary (b) amplitude holograms, and gray-scaled (c), binary (d) and blazed (e) phase holograms. The efficiency is related to the amplitude of the modulation A, which varies between 0 and 1 in the case of amplitude holograms and between 0 and 2π in the case of phase holograms. 

The efficiency of (a) and (b) reaches, in the best conditions, 6.2% and 10.1%, respectively, while for phase hologram (c) and (d) the efficiency is 34% and 41% respectively. In the case of the blazed hologram, it can even reach the 100% efficiency.

In the case of photochromic materials, both amplitude and phase holograms are possible, according to the working region of the hologram. For amplitude holograms, the parameter A is directly linked to the contrast between transparent and opaque regions, i.e., to the transmission of the photochromic film in the transparent and colored forms. The contrast, which is a wavelength dependent quantity, in a region where only one of the two forms is fully transparent, is given by the dye concentration *C*, the molar absorbance *ε* of the colored form, and the thickness of the film *d* as follows:

(5) $$Contrast=\frac{T_{transparent}}{T_{coloured}}=\frac{1}{10^{-Abs}}=10^{\varepsilon Cd}$$

**Figure 2:**
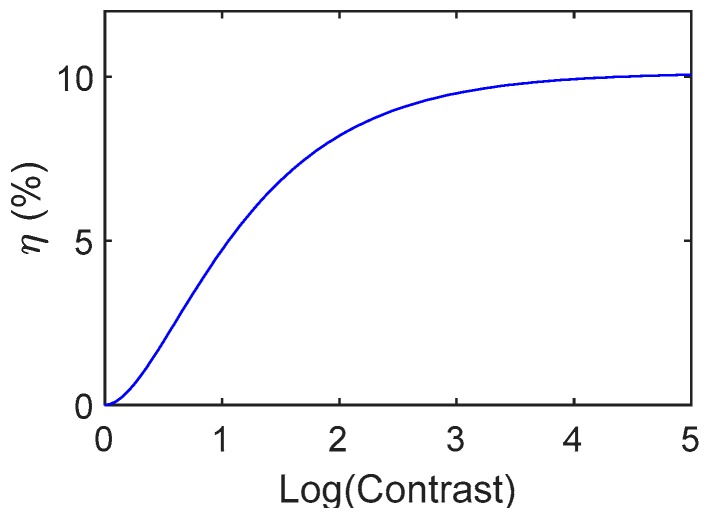
First order diffraction efficiency of a binary amplitude grating as function of the contrast value [45].

Figure 2 reports the first order diffraction efficiency of a binary hologram as function of the film contrast [45]. 

The contrast is asymptotic to the maximum efficiency of 10.1% for values larger than 5000, but values around 8% are good enough for many applications and in particular for interferometric purposes. These efficiencies are reached when the contrast is larger than 100, value that is obtained for optical density of the film in the colored form larger than two (considering again an absorbance zero for the uncolored form).

**Figure 3:**
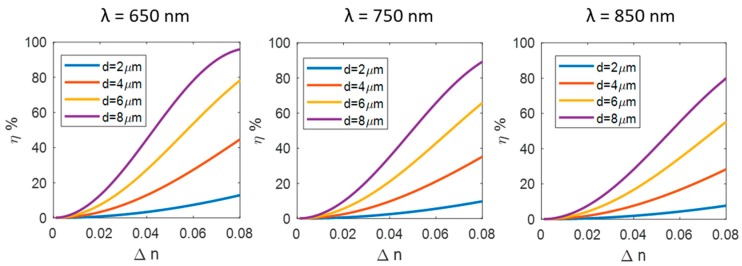
Theoretical efficiency of phase gratings as function of the refractive index modulation for films with a thickness of 2, 4, 6 and 8 µm. The results are shown for three different wavelength 650, 750 and 850 nm; the grating line density is 1000, 870, 770 lines/mm, respectively.

Concerning phase holograms, the key parameter to deal with is the product between the refractive index modulation and the film thickness d·Δn. For example, if we take the efficiency of volume phase gratings working in the Bragg regime, we can write the first order diffraction efficiency at the Bragg angle ($\alpha_{B}$) as [46]:

(6) $$\eta =\frac{1}{2}\sin^{2}\left(\frac{\pi \Delta nd}{2\lambda cos\alpha_{b}}\right)+\frac{1}{2}\sin^{2}\left(\frac{\pi \Delta nd}{2\lambda cos\alpha_{b}}\cos\left(2\alpha_{B}\right)\right)\;$$

where *λ* is the wavelength of the incident light. We studied the efficiency dependence by Δ*n*, considering λ=650nm,750nm, 850 nm and $\alpha_{B}=$ 19°. In Figure 3, we report the results for a Δ*n* in the range 0–0.08.

Considering that diarylethene based photochromic films can reach Δ*n* of 1–4%, we are able to write phase binary gratings with good efficiency, depending on the film thickness. However, the maximum useful thickness of the photochromic materials is limited by the UV penetration (more details are provided later on), which determine the degree of conversion through the film thickness. Therefore, we can conclude that there is a sort of upper limit in the d·Δn value for the photochromic films.

## 3. Diarylethenes: Properties Modulation

**Figure 4:**
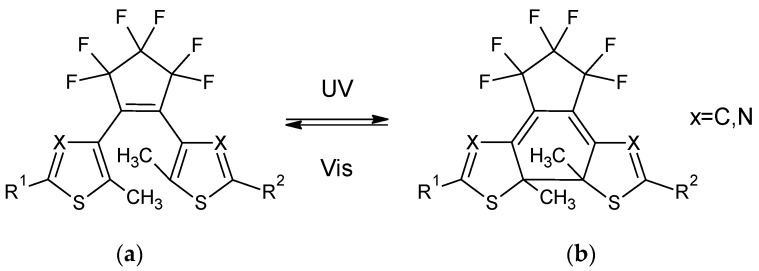
Photoreaction in 1,2-diarylethenes considered in this work. (**a**) open form (uncolored); (**b**) close form (colored). The detailed structures are reported in Figure 5.

Diarylethenes show a light-induced transformation between two forms a and b as reported in Figure 4 in the case of the perfluorocycplopentene derivatives. The a form, called open form, is usually uncolored since the π-conjugation is interrupted between the two side parts of the molecule. Upon illumination with UV light, a 4n + 2 electrocyclization occurs, and the b form, called close form, is obtained. This state is characterized by a π-conjugation extended along the whole molecular backbone, with a consequent coloration of the materials.

Actually, hundreds of diarylethenes have been synthesized so far, and comprehensive reviews report on the main characteristics and possible applications of this important family of photochromic compounds [47,48]. In this review, we limit the discussion to a series of diarylethenes, and we discuss the modulation of absorption properties in the UV-Vis as a function of the chemical structure specifically to later highlight the conversion in the film state, which is of fundamental relevancy to reaching an adequate contrast in amplitude holograms. Moreover, for possible application as phase holograms, features for maximizing the refractive index modulation are reported.

### 3.1. UV-vis Absorption

**Figure 5:**
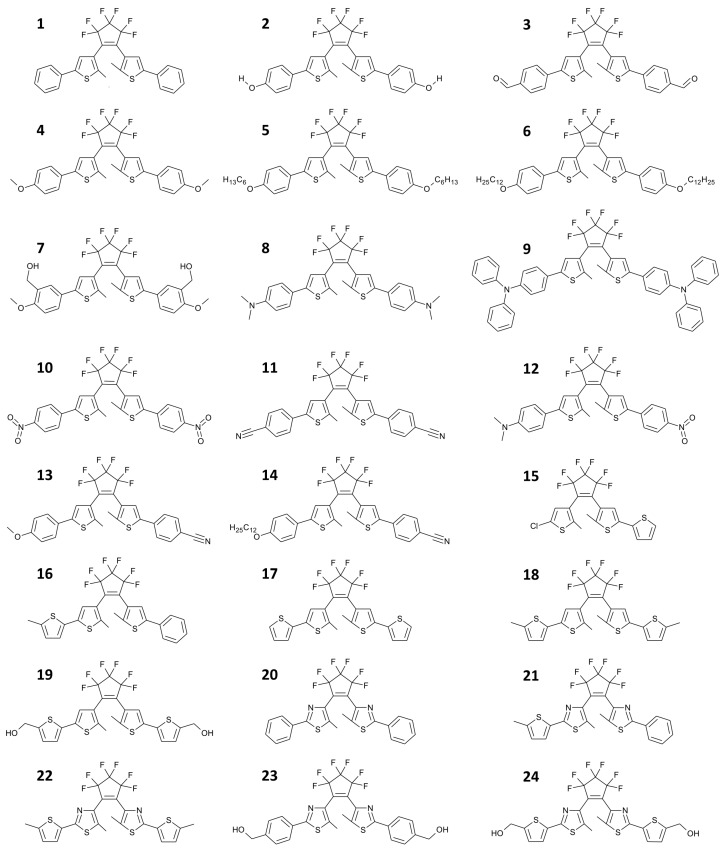
Series of 1,2-diarylethenes in their open state (uncolored). They differ for the aromatic ring in the switching structure (thienyl from **1** to **19** or thiazolyl from **20** to **24**) and for the lateral groups. Electroactive substituents can be also present to give push-push structures (compounds from **4** to **9**), pull-pull structure (compounds **3**, **10** and **11**) or push-pull structures (compounds from **12** to **14**) in their closed (colored) state.

In a liquid solution, the photochromic process approximately occurs uniformly in the whole volume, and the conversion at the photosteady state depends on the absorption coefficients ($\varepsilon_{A},\varepsilon_{B}$) of the two isomeric forms at the irradiation wavelength and on the quantum yield of forward and backward reactions ($\phi_{AB},\phi_{BA})$. All these quantities depend on the molecular building blocks, both those ones involved in the 4n + 2 electrocyclization (i.e., the photoactive part of the molecule) and the lateral substituents. Many diarylethene derivatives have been synthesized so far, and the effect on the specific chemical structure on the absorption properties for a selection of compounds (see Figure 5) is highlighted in Table 1.

**Figure 6:**
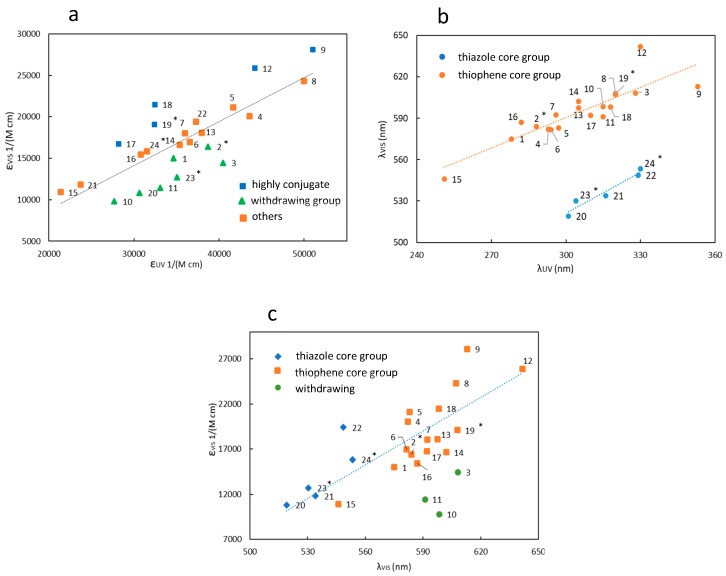
(**a**) Plot of the molar extinction at the maximum absorbance in the visible of the colored isomer $\varepsilon_{vis}$ and in the UV of the transparent isomer $\varepsilon_{UV}$, for the diarylethenes of 5; (**b**) Plot of the wavelength of the visible peak for the colored isomer and for the UV peak of the transparent isomer; (**c**) Plot of the position and the molar extinction coefficient of the band in the visible for the colored isomers. Molecules were characterized in hexane except for * which were dissolved in ethanol.

Despite the fact that the values reported in Table 1 seem to be highly scattered, correlations between the different parameters can be found for the different groups of diaryethenes herein synthesized and analyzed. The comparison between the molar extinction coefficients of the uncolored and the colored forms of any diarylethene ($\varepsilon_{UV}$, $\varepsilon_{VIS}$, respectively) shows that the maximum absorbance of the visible band of the colored isomer is roughly half of the absorbance in the UV (Figure 6a). Moreover, diarylethenes with lateral substituents characterized by the presence of a phenyl group, either alone or linked with a withdrawing functional group, have a lower intensity of the visible band (green series). Indeed, all of these molecules have a less-conjugated structure in their colored forms. Conversely, molecules belonging to the blue series have a more intense visible absorption, which can arise from all the possible different chemical structures allowing for an extended π-conjugation in the closed form, e.g., the use of thiophene-thiophene as lateral substituent (compounds from **17** to **19**) and the push-pull substituents (compounds from **12** to **14**). In addition, the triphenylamine as substituent is known to give an effective π-conjugation (herein compound **9**).

The analogous analysis, but considering the absorption maxima instead of the absorption coefficient, leads to the general conclusion that a redshift of the visible band of the colored form corresponds to a redshift of the UV band of the uncolored form (Figure 6b). The two series of data in the figure correspond to the dithienylethenes (orange data) and the dithiazolylethenes (blue data). For both series, the wavelength gap between the absorption maxima of the colored and uncolored forms is approximately the same inside the members of the same series. In particular, the gap is about 220 nm for the thiazole based series and 290 nm for the thienyl based one. Actually, the presence of electroactive substituents can modify this wavelength gap, i.e., push-pull substituted dithienylethenes (compounds from **12** to **14**) are characterized by a larger λ_VIS_-λ_UV_, with the largest difference value for the compound **12**, having both very strong donor and acceptor groups.

Finally, the relationship between $\varepsilon_{vis}$ and λ_VIS_ is highlighted (Figure 6c), since the behavior of the photochromic molecules in the visible (i.e., the contrast) is relevant for the development of amplitude holograms. The overall evidence is that the longer the wavelength of the peak, the higher its absorption intensity [49], which is a common trend in conjugated molecules [50]. However, the presence of withdrawing groups (e.g., compounds **9** and **10**) decreases the absorption coefficient, whereas donor groups lead to higher absorbance (compounds **4**–**9**).

At the solid state, including dyes in the crystalline or amorphous state and polymer dispersed dyes, the situation is more complex since the light-induced process proceeds from the outer layer to the inner layer. Actually, the full transformation from the colored to the uncolored forms is always possible, since only the colored isomer absorbs in the visible. Instead, the coloration process is not straightforward as both the isomeric states of diarylethenes absorb UV light. Therefore, the radiation is attenuated through the volume while the coloration proceeds and a limit depth of UV penetration exists, beyond which the photochromic reaction cannot further occur. In addition, the degree of conversion through the thickness follows a gradient depending on the illumination time [45]. In this condition, the measurement of molecular absorption properties (ε, φ) is tricky. Nevertheless, it is still possible, by considering the local degree of conversion of the molecules inside the film or by using very thin films where the conversion can be considered uniform. 

**Figure 7:**
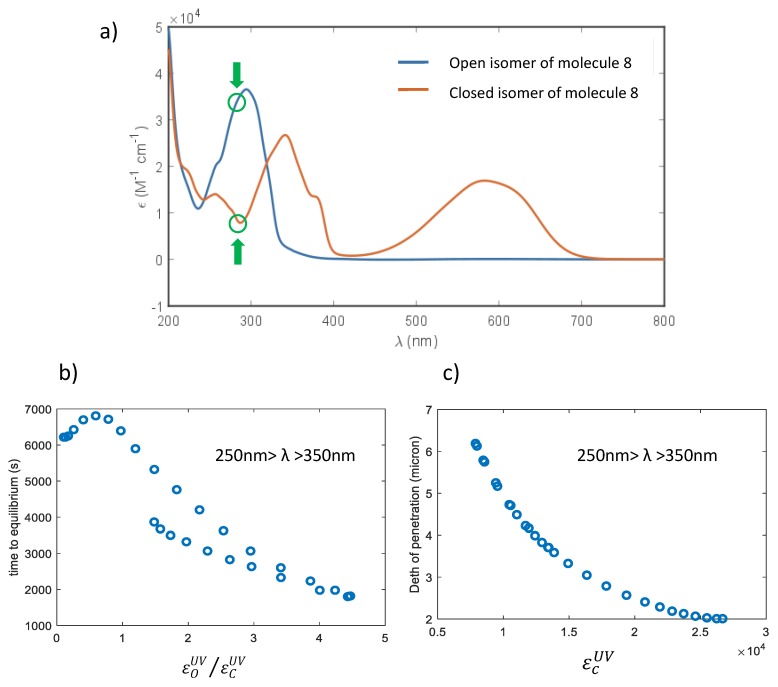
(**a**) UV-vis absorption spectra of the two isomers of molecule **6**; (**b**) time necessary to reach the photostationary state as function of the ratio $\varepsilon_{O}^{UV}/\varepsilon_{C}^{UV}$; (**c**) penetration depth as function of the irradiation wavelength. With the green circles the values of the molar extinction coefficients at the wavelength corresponding to the highest penetration are highlighted. Each point of figure (**b**) and (**c**) corresponds to a different simulation with a λ of irradiation changing between 250 nm and 350 nm.

Supposing that the transparent form absorbs more than the colored one in the UV range of illumination, the UV penetration at the end of the conversion is determined by $\varepsilon_{C}^{UV}$, namely, the extinction coefficient of the colored form in the UV. In Figure 7, the case of a 10 μm thick film with a concentration of 400 mol/m^3^ of molecule **6** is reported, showing the measured absorption spectra of the two forms and the calculated penetration depth as a function of the molar extinction coefficients.

The lower the absorption of the UV light by the colored form, the higher the penetration (Figure 7c). Fixed the quantum yield of the transformations ($\phi_{CO}^{UV},\phi_{OC}^{UV}$), the time required to reach a stationary situation decreases while increasing the ratio $\varepsilon_{O}^{UV}/\varepsilon_{C}^{UV}$. It has been also demonstrated that this ratio affects the fatigue resistance of the diarylethenes [51].

**Figure 8:**
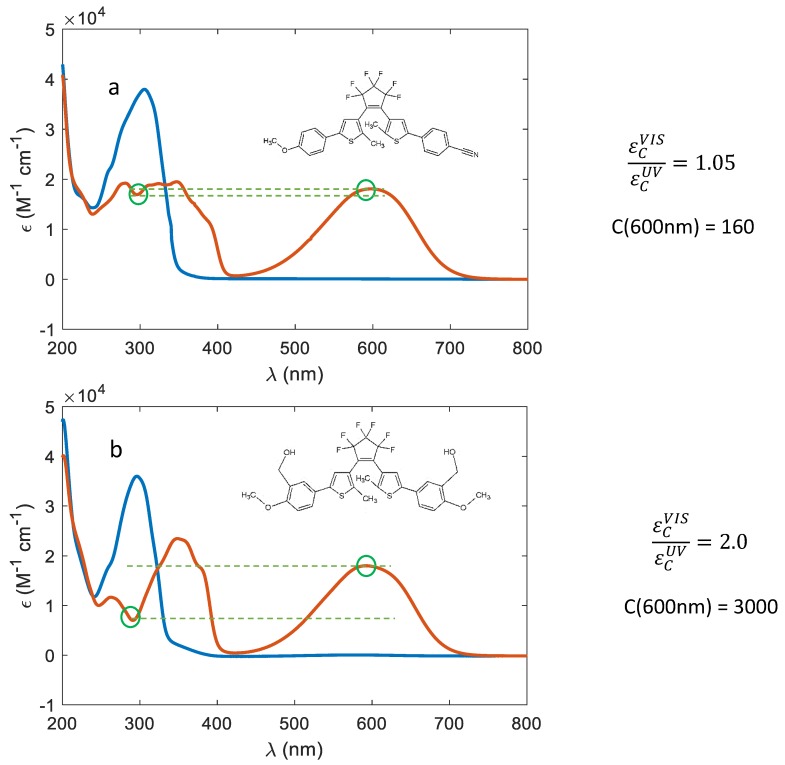
Comparison between two diarylethenes showing a different $\varepsilon_{C}^{vis}/\varepsilon_{C}^{UV}$ ratio: (**a**) Molecule **13**; (**b**) molecule **7**. On the left the molecules used in the simulations, together with their absorption properties are reported (the green circles highlight $\varepsilon_{C}^{UV}$ and $\varepsilon_{C}^{vis}$); on the right, the value of the ratio $\varepsilon_{C}^{vis}/\varepsilon_{C}^{UV}$ and the contrast C computed at 600 nm are reported for a 10 μm film with a concentration of 300 mol/m^3^.

All these considerations point out that the actual coloration of a photochromic material at the solid state depends not only on the intrinsic capability of absorbing visible light by the colored form, but also on its absorption at the illumination wavelength. This means that to reach large contrasts, large $\varepsilon_{C}^{vis}$ cannot be the only selection criteria of a photochromic dye. If the absorption at the illumination wavelength ($\varepsilon_{C}^{UV}$) is high and comparable to the $\varepsilon_{C}^{vis}$(highlighted in Figure 8a for compound **13**), the penetration depth will be low, and the contrast will be similarly visible (C = 160). Instead, if the absorption is much lower, a consistent raise of the contrast value results (C = 3000 for compound **7**, Figure 8b). 

### 3.2. Computational Tool

Once we had determined the relevant parameters that characterize the general photochromic behavior at the solid state, we made use of a kinetic model which describes both the coloration and the fading of a diarylethene film under specific illumination conditions [52], and we combined here all these pieces of information in a computational tool, which predicts its performance a priori. This allows for a proper selection of the photochromic material, which is necessary to satisfy the target properties of the optical elements (both phase and amplitude holograms), without a number of optimization experiments that would have been otherwise necessary, saving time and material. 

In order to efficiently exploit the kinetic model, a Graphical User Interface (GUI) using Matlab^®^ R2016b (The MathWorks, Natick, MA, USA) was built, facilitating the selection of the simulation parameters and displaying the desired results with a fast and practical routine. GUI is user-friendly, hence it can be used without any specific computational ability. 

Two different versions of the tool were developed, enabling to simulate a photochromic film based on either one or two molecules, mixed together. In the following, the two molecules case is detailed, being the most complex one. 

**Figure 9:**
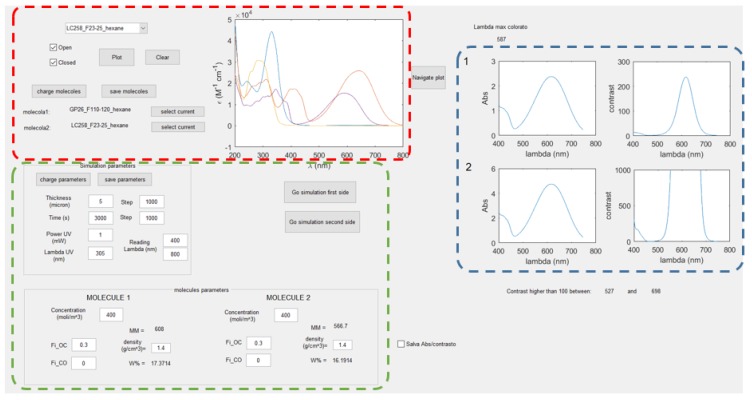
Screenshot of the GUI developed to simulate the absorption properties of photochromic films. The colored boxes indicate three different sections: selection of the molecules (red), parameters choice (green) and results visualization (blue).

The program is organized in three different sections: (i) selection of the molecules, (ii) parameters choice and (iii) results visualization (Figure 9).

#### 3.2.1. Selection of the Molecules

The absorption properties in solution or solid state of the transparent and colored isomers (e.g., molar extinction coefficient as function of the wavelength) of a series of dyes are previously uploaded in a database. In this window, the UV-vis absorption spectra of one or two dyes picked up from this database are shown, allowing for an easy and rapid comparison (Figure 9 red box). 

The key features usually considered are: (i) the wavelengths at which the photochromic transformation can be triggered; (ii) the correspondent absorbance values and the position and width of the absorbance band in the visible. Considered together, they give hints about the efficiency of the transformation and the possible final behavior of the materials. 

#### 3.2.2. Parameters Choice

In this window, the illumination conditions, including the wavelength and the illumination intensity, and the film thickness are set (Figure 9 green box). Setting the first parameters, the discretization of time and space is defined. In this section, we also set the wavelength range where the results will be computed. 

On the material regard, the concentration of the selected molecule and its photochromic properties are selected, specifically the quantum yield of conversion at the illumination wavelength for the direct ($\phi_{OC})$ and inverse ($\phi_{CO})$ transformation, and the density of the material (usually considered equal to the density of the chosen polymer matrix). In the case of two dyes, the concentration of molecule 1 and molecule 2 is set.

#### 3.2.3. Visualization of the Results

**Figure 10:**
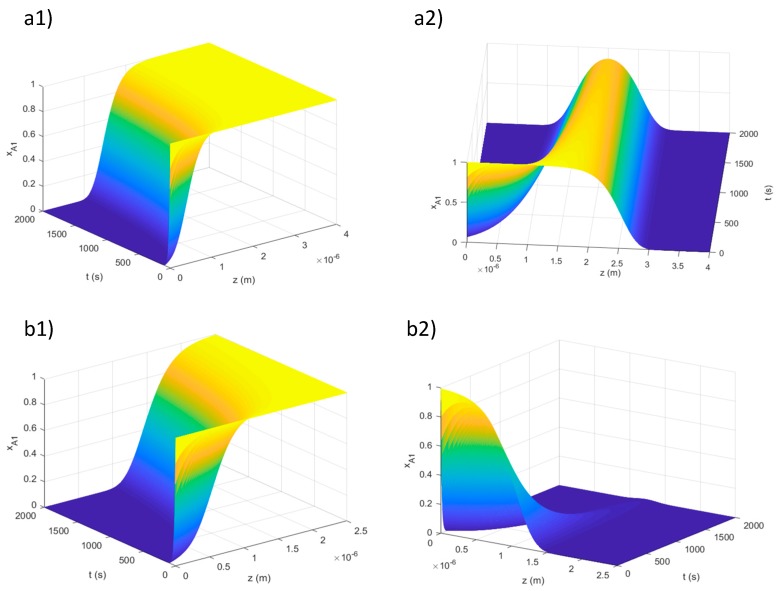
Concentration profile of the transparent isomer after the simulated irradiation of one (**a1**,**b1**) and two sides (**a2**,**b2**). The plots show the time evolution of the profile and the penetration inside the volume. In this example, we show a film of 4 μm, where it is impossible to convert all the material (**a2**), but lowering the value to 2.5 μm means the total conversion is reached after the two side illumination (**b2**). Photostationary state after 2000 s.

Once we had selected the molecules and the simulation parameters, the computation could start. The program is divided into two steps, enabling us to simulate a double illumination process, with one process for each side of the film. At each stage, the concentration profile of the transparent isomer is plotted, as function of time and space, and the limit of the UV penetration is computed. By the analysis of these plots, it is possible to understand if either the molecular concentrations or the film thickness is too large to have a complete conversion inside the sample volume. In Figure 10, we show examples where the total conversion inside the film volume is reached or not, depending on the material thickness. The plots show the transparent isomer concentration in dependence of time and thickness, after one (a1, b1) and two (a2, b2) sides were illuminated. 

In the case reported in Figure 10a1, we notice that a large amount of the film is not converted by a single side illumination. Also with a double side illumination (Figure 10a2), the inner part of the film remains unconverted. In the case b, a single side exposure is not enough to achieve the total conversion, but a double illumination (Figure 10b2) induces a complete coloration.

**Figure 11:**
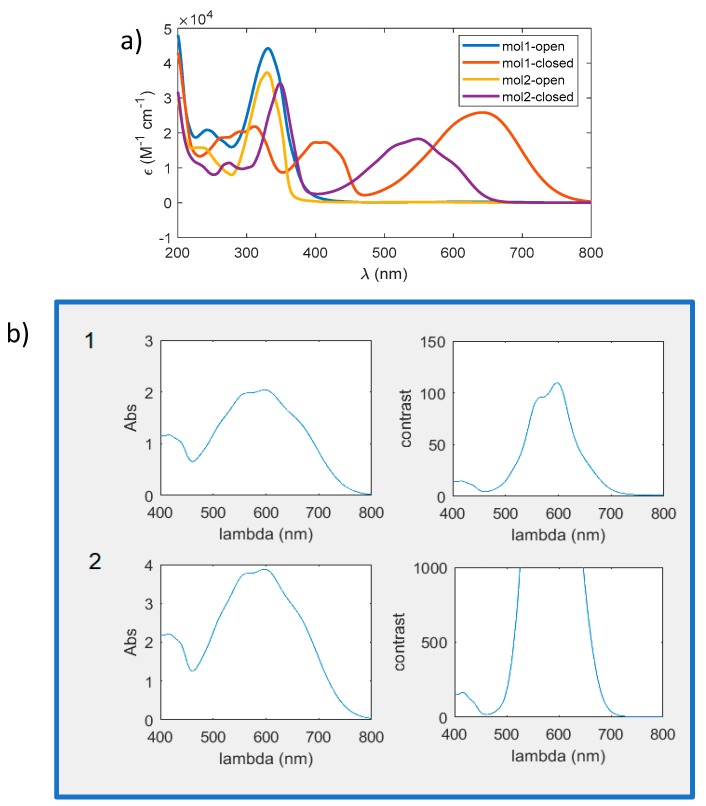
(**a**) Molar extinction coefficient of two diarylethenes (molecule **12** and **24**) used for the simulation; (**b**) Screenshot of the results computed: the figure shows absorbance and contrast of the film after one and two exposures (performed on two sides) in the spectral range of interest.

In Figure 11, we report an example of how the absorbance properties of two different dyes can be combined to reach higher contrast performances.

In this case, two molecules with a visible absorbance around 550 nm and 650 nm are combined to cover a wide wavelength range. In panel b), we report the absorbance and relative contrast of the film, after one and two sides exposure considering a thickness of 4 µm and a concentration of about 16 wt % for both dyes.

We notice that the absorbance almost double going from one side to the both sides illumination, meaning that the penetration depth is roughly half of the film thickness. As for the contrast (plots on the right), the increase is very large by the double exposition, reaching values larger than 1000 in a range wider than 100 nm. We also notice that in the 400 nm region, the contrast is quite good (>100) thanks to the presence of the secondary peak in mol1 (Figure 11a) and only a small spectral region around 450 nm has a low absorbance. 

### 3.3. Remarks on the Absorption Properties 

According to the discussions we reported, it is clear how useful such tool can be in designing high performance photochromic films and how many pieces of information can be retrieved from the simulations. Given a desired contrast in a certain wavelength range for a specific application, this tool supports the choice of the right set of molecules to be used. Once they have been selected, the illumination wavelength has to be carefully chosen: $\varepsilon_{c}^{UV}$ should be low, to have a deep penetration through the film, thus achieving a full conversion; moreover, the $\varepsilon_{O}^{UV}/\varepsilon_{C}^{UV}$ and $\varepsilon_{C}^{vis}/\varepsilon_{C}^{UV}$ ratios should be high in order to have a fast kinetic and high contrast. 

In the case of a mix of two dyes, the choice must be particularly careful. Both molecules have to be efficiently converted at the same time with the same illumination wavelength, meaning that they should have similar absorbing profiles in the UV region. Otherwise, one dye could behave as a barrier for the conversion of the other one, resulting in lower and unexpected absorption performances. 

Finally, information on the concentration of the dye and the film thickness are provided to fulfill the requirement of contrast. With the proper selection of the diarylethene and films of few microns of a polymer matrix containing 20–25% of chromophore or backbone photochromic polymers, it is possible to reach a suitable contrast for the application herein reported.

### 3.4. Refractive Index Modulation

In the case of volume phase devices, we showed that a modulation of the refractive index is necessary in order to induce a controlled phase delay, which is equal to the product refractive index *n* times the film thickness *d*. Considering a target product d·Δn of about 0.4–1 μm, values of Δn = 0.04–0.1 are required for film thicknesses in the range of 1–10 μm.

In order to understand this requirement from a materials point of view, we start from the Lorentz-Lorenz equation. This equation [53] links the macroscopic refractive index with the material density and the molecular polarizability α (since we are in the optical spectral region only electronic polarizability is considered):

(7) $$N=\frac{n^{2}-1}{n^{2}+2}=\frac{4\pi }{3}\frac{\;N_{A}}{V}\;\alpha$$

where *n* is the material refractive index, $N_{A}$ the Avogadro number, V is the molar volume. This is valid for a monocomponent material, but photochromic films are multicomponent and the refractive index contains a contribution of both the matrix and the photochromic dye. Considering no interaction between these two components, we can write an effective refractive index as the sum of their contributions:

(8) $$\frac{n^{2}-1}{n^{2}+2}=C_{matrix}\frac{n_{matrix}^{2}-1}{n_{matrix}^{2}+2}+C_{dye}\frac{n_{dye}^{2}-1}{n_{dye}^{2}+2}=C_{matrix}N_{matrix}+C_{dye}N_{dye}$$

(9) $$C_{matrix}+C_{dye}=1$$

where $n_{dye},n_{matrix}$ are the refractive indices of a material composed by the pure dye and the polymer matrix respectively and $C_{matrix,}C_{dye}$ are the relative volume concentrations. Accordingly, the refractive index of the colored (or uncolored) material is then:

(10) $$n_{c\left(o\right)}=\sqrt{\frac{2C_{dye}\left(N_{dye}^{c\left(o\right)}-N_{matrix}\right)+2N_{matrix}+1}{1-N_{matrix}-C_{dye}\left(N_{dye}^{c\left(o\right)}-N_{matrix}\right)}}$$

We noticed that the refractive index of the film depends on the contrast between the value of the matrix and of the photochromic dye. Usually, the matrix shows a refractive index lower than the value of the photochromic dye. Even more important for determining the refractive index of the doped film is the concentration of the photochromic species. It must be as large as possible, but avoiding any side effects such as segregation or aggregation. Actually, we are interested in the change in the refractive index going from one photochromic form to the other. Looking again to the Equation (7), we notice that a large change in the molecular polarizability α between the two forms is necessary, in addition to the previous requirements, to enhance the modulation in the refractive index. The polarizability is proportional to the number of electrons in the molecule, but it is known that π conjugated systems exhibit higher polarizability, and the enhancement is proportional to the degree of delocalization [54]. Considering diarylethenes, it is apparent that the closed (colored) form is more conjugated than the open (uncolored) form; therefore, it shows a larger refractive index. The presence of electroactive substituents can play a role in increasing the modulation of the refractive index [55]. Moreover, the molecular polarizability is wavelength dependent as the refractive index. In the optical regime, it increases with the frequency in a marked way approaching the resonance frequencies due to the electronic transitions [56]. Since the colored form shows visible absorption bands, this pre-resonance effect will be more important than for the uncolored form (only UV absorptions). Moreover, a steeper increase in the molecular polarizability takes place in the NIR. Consequently, the modulation of the refractive index will benefit from this effect and it increases with the frequency too [57]. This feature has been recently highlighted [49] in a series of diarylethene based polyurethanes, where a clear positive trend existed between the Δn and the absorption wavelength of the colored form. 

To sum up, in order to maximize the modulation, it will be necessary to:

- maximize the concentration of the photochromic dye in the film;
- design a photochromic molecule with specific chemical groups that enhance the change in the molecular polarizability (large change in the π conjugation path and efficiency);
- increase the wavelength gap between the absorption band in the UV of the uncolored form and the visible band of the colored form.

According to the experimental results reported in the literature, the concentration parameter is the most important one in affecting the Δn and for these reasons, backbone photochromic polymers have been developed. Values of the order of 0.08 at 800 nm were measured [58].

## 4. Writing Strategies and Examples of CGHs

In a typical route for the CGH production, the photochromic film is converted to the colored form by irradiation with UV light. Then, the layer is patterned upon exposure to visible light, which induces a selective bleaching of the film. We considered two different strategies here for the substrate patterning: (i) a mask projection system, based onto a spatial light modulator; (ii) a scanning system, by direct laser writing (maskless lithography). The two techniques, presented hereafter, are complementary. In both cases, the writing process may not introduce imperfections, called pattern distortions, especially when the holograms are used in interferometric applications. They are basically due to a misalignment of the writing beam with respect to its ideal position and can be quantified as the introduced wavefront error $\Delta W_{\zeta }$ [59]:

(11) $$\Delta W_{\zeta }=-\frac{m\lambda \zeta }{G}$$

where *m* is the diffraction order, ζ  is the grating position error in the direction perpendicular to the pattern lines and *G* is the local line spacing. To minimize these errors, it is convenient to work at low diffraction orders and with coarse line patterns. Along with this, the quality of the reconstructed image depends on the planarity of the substrate, since any imperfection produces phase contaminations. This is valid for the substrate itself, as well as for the photochromic film. It is crucial, accordingly, to optimize the depositing process not to introduce high spatial frequency errors in the transmitted wavefront, for both the film thickness and planarity. 

### 4.1. Mask Projection

**Figure 12:**
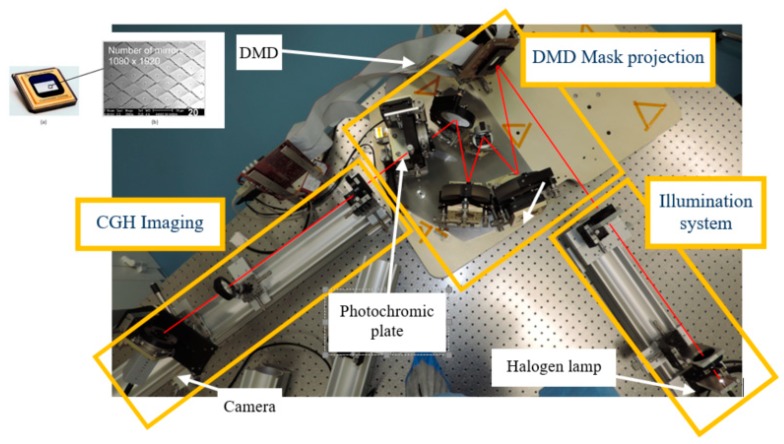
DMD based set-up for writing the photochromic CGHs. The three main subsystems, namely illuminating system, DMD mask projection and CGH imaging are highlighted. In the inset, a picture of the DMD used is reported [61].

This approach consists in the projection of a mask specifically designed with the target pattern onto the photochromic substrate. Such approach derives directly from the well-established mask lithography [60]. An interesting possibility consists of the image projection through an Offner relay, which produces a one to one projection of the mask plane, where a Digital Micromirror Device (DMD) is placed, onto the sample plane, where the photochromic film is [61]. A DMD is a rectangular pattern of micromirrors that can be independently addressed between two specific angular positions. The device used in our tests by Texas Instruments (Dallas, TX, USA), is composed by 2048 × 1080 micro-mirrors with a pitch of 13.64 µm. The optical quality of the system is limited by the micromirror size and not by the optical aberrations. During the writing process, the DMD is homogeneously illuminated by a filtered light source. A CGH imaging system is also present, to follow the writing process in real time (Figure 12). 

**Figure 13:**
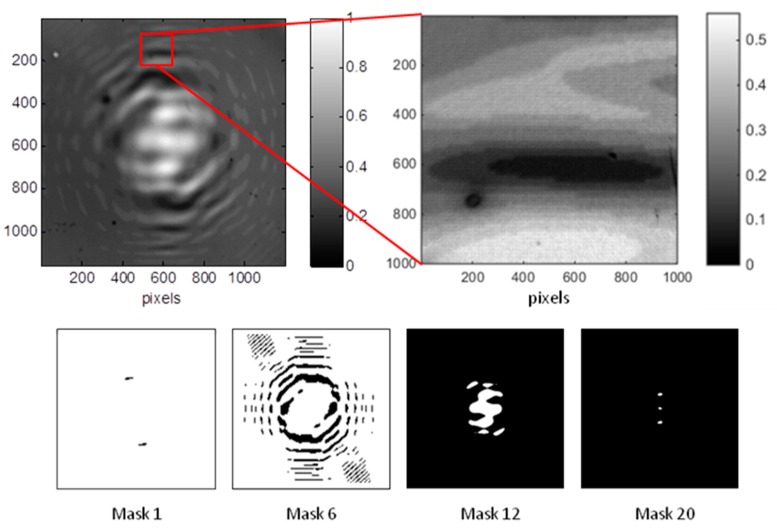
A grayscale CGH, the magnification shows the well-defined gray levels; examples of masks used for its production [62].

Recording a binary CGH requires the projection of a single DMD mask for enough time to produce the full conversion of the film from the opaque to the transparent form. The advantage of the DMD projection system is the possibility to easily write grayscale CGHs [62]. Since the DMD is a programmable device, any mask can be projected for a specific amount of time. In fact, the photochromic material becomes progressively transparent when illuminated by visible light, and a given level of transparency, i.e., a given level of gray, is obtained with a well-defined exposure time. Caution is needed since the material response is not linear with the exposure time, but the transmission curve as function of the expose time can be measured before the CGH production and used for the linearization. Figure 13 illustrates an example of a grayscale CGH, with four different masks used for its realization. 

The versatility of the DMD is the great advantage of this technique: although a binary amplitude CGH has a higher efficiency, a grayscale hologram enables for much better image reconstruction quality, which leads to a better control on the wavefront generated by the CGH. On the other hand, the image resolution is limited by the micromirror size and number, i.e., by the ratio between the dimensions of the single micromirror and the whole chip. A possible step forward could be the stitching of different DMD projections to create a larger CGH or to demagnify the DMD, thus increasing the resolution (the image of the single mirror is smaller) and then stitching the different DMD images.

### 4.2. Direct Laser Writing

With direct laser writing, the pattern is transferred to the photosensitive layer using a light beam focalized in a theoretically diffraction limited spot onto the substrate. The laser power can be continuously adjusted while the substrate is scanned in the plane and exposed where necessary. Usually, an autofocus system keeps the substrate in the correct axial position to guarantee the best spot resolution. In the past, we investigated the possibility to use commercial direct laser machines to transfer patterns onto photochromic substrates [63], but we faced problems due to the writing speed, light power, and wavelength. In fact, commercial systems are characterized by high speed rates (hundreds of mm/s), very high light powers (tens of mW/μm^2^), and usually work in the spectral region suitable for photoresists, namely in the UV, which is not really suitable for diarylethenes. We observed a low definition of the pattern, and the formation of surface reliefs on the coating given by the local heating of the substrate. In contrast, the resolution was very high, being limited by the spot size (down to 1 μm). We therefore developed custom direct laser machines for the production of photochromic CGHs, where we optimized the writing speed, the light power and the writing wavelength. 

**Figure 14:**
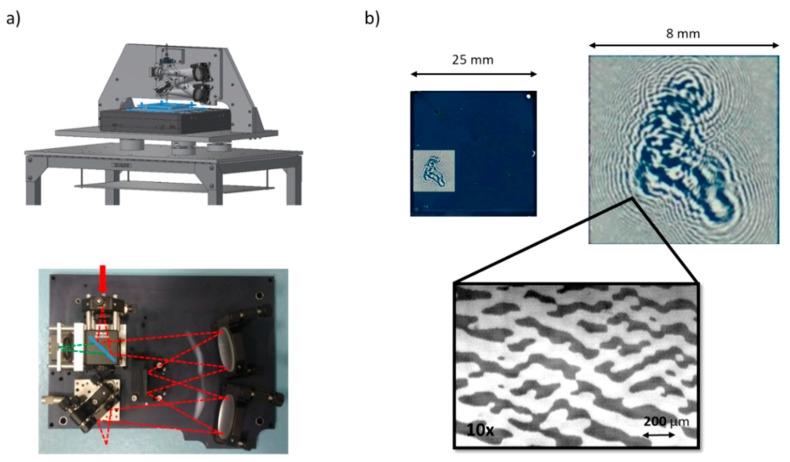
(**a**) Mechanical (top) and optical (bottom) schemes of our direct laser writing machine; (**b**) Different magnifications of a typical sample of photochromic film, written with a Fresnel CGH [64].

The developed system is shown in Figure 14 [64]. It is composed by a moving table (raster X-Y scan) and an optical bench (Offner relay layout), mounted vertically on a fixed bridge. The light source is a multichannel laser system equipped with four heads at 406 nm, 520 nm, 638 nm and 685 nm. The different wavelengths were selected as function of the sensitivity curve of photochromic materials and can be used independently. The light is coupled to an optical fiber and guided to the writing head. A trigger mechanism driven by the linear stage switches the lasers on and off at MHz speed. A viewing camera is also present to align the substrate and follow the writing process. The spot size is 3–4 μm depending on the wavelength, the writing speed 1–3 mm/s, and the laser power at the focal plane 1–3 mW. 

### 4.3. Examples of Diarylethene-based CGHs

Here we show some examples of holograms obtained with photochromic films based on diaryletehenes. As previously discussed, the calculated hologram phase function can be approximated as an amplitude or phase pattern, both binary and grayscale, and transferred to the photochromic layer with the most appropriate technique. While for binary CGHs direct laser writing is preferred, grayscale CGHs can be more easily obtained with the mask projection technique. In the latter case, recording a binary CGH requires the projection of a single mask to the photochromic plate, while grayscale holograms can be obtained by sequentially displaying a series of binary masks to locally create the desired level of transparency [61]. Considering diarylethenes, amplitude holograms performs well in the visible region, approximately between 500 and 800 nm, while phase holograms performed well in the NIR region, approximately between 800 and 1500 nm. 

**Figure 15:**
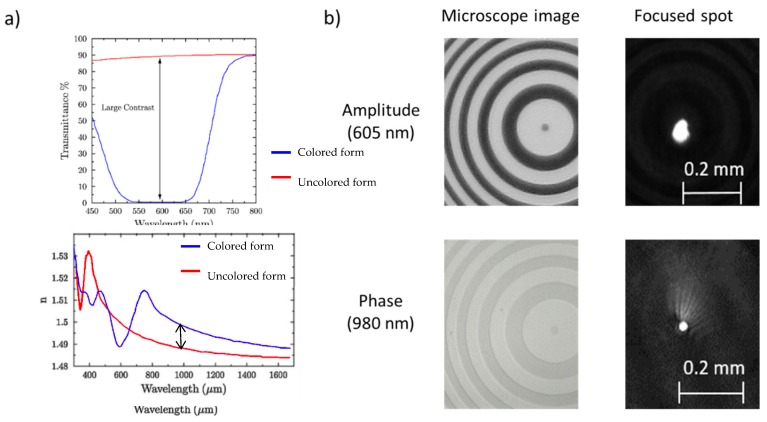
(**a**) Measured modulation of transmittance and refractive index of a high content photochromic film; transparent form (red line) and opaque form (blue line). The arrows highlight the change in the transparency and refractive index; (**b**) Left: Microscope images (phase and amplitude) of a Fresnel CGH recorded on a diarylethene based film. Right: CCD images of the laser spot in the focal plane [49].

A nice example is the CGH of a Fresnel lens reported in Figure 15 [49]. This CGH behaves as a spherical lens and the focal length is dependent on the spacing of the lines. As clearly shown in Figure 15b, it is possible to identify a focused spot on the camera both illuminating the CGH with a red laser (650 nm), where the hologram behaves as an amplitude hologram, and illuminating the CGH with a NIR laser (980 nm), where the hologram is a pure phase hologram. The corresponding transmission spectra and the refractive index dispersion curves are reported in Figure 15a, where it is marked with arrows showing the change of property between the two forms in the film. 

**Figure 16:**
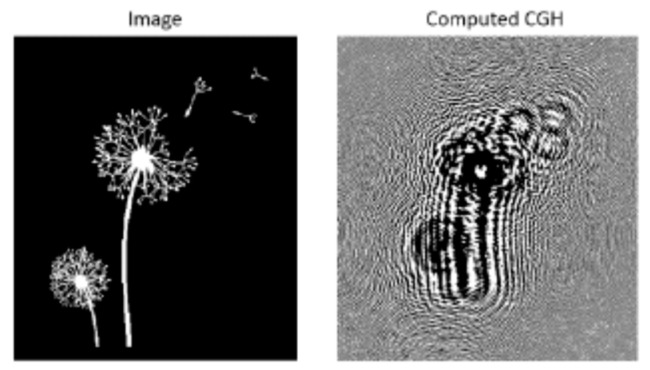
Image of a dandelion and the calculated binary Fresnel CGH (4 × 4 mm^2^ size at a focus of 0.5 m) [64].

Another example of photochromic CGHs is reported in Figure 16. It is the image of a dandelion (430 × 430 pixels), that has been transferred by direct laser writing. The hologram is a binary amplitude type, square with a 17 mm side, and a pixel size of 3 μm.

**Figure 17:**
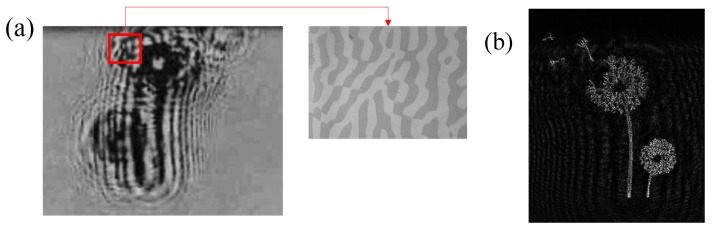
(**a**) Photograph of the CGH and a magnification of the written pattern; (**b**) The reconstructed image at 633 nm [64].

The calculated CGH was transferred on the photochromic film by means of the direct laser writing machine and the result is reported in Figure 17 details of the pattern. 

The image is reconstructed at 0.5 m, with a size of 4 × 4 mm^2^ (Figure 17b). We notice the complexity of the image with small details, which requires a large hologram due to the high information density on its edge. Hologram resolution and size prevented the use of the mask projection technique to obtain the same level of details.

The second example is the image of a letter “Z” (200 × 200 pixels), obtained by mask projection. Accordingly, the hologram is grayscale amplitude. The size of the CGH is limited to 10 × 10 mm^2^, which leads to a CGH resolution of 720 × 720 pixels according to the DMD size. In order to be sure that all the fringes in the CGHs are resolved, the image physical size and the focus are fixed at 2 × 2 mm^2^ and 2 m, respectively. Once we obtained the continuous complex pattern, its magnitude was discretized to twenty gray levels with thresholds ranging from 0 to 1 in steps of 0.05. 

**Figure 18:**
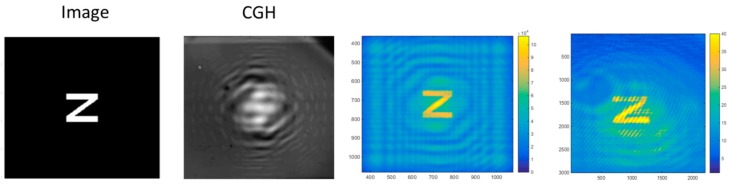
From left to right: the calculated and recorded (grayscale) CGH on the photochromic polymer; simulated and experimentally reconstructed image at 633 nm [62].

Figure 18 shows the calculated and the actual grayscale CGH of the letter Z, along with the theoretical and experimental reconstructed image. 

The image, reconstructed at 633 nm, shows a dimension on the camera of 2 × 2 mm^2^, as expected. Very faithful reconstruction has been obtained with respect to the simulated reconstructed image as well as the original “Z” image. Also in this case, we can notice the fidelity of the reconstruction, confirming the effectiveness of the mask projection approach to produce amplitude grayscale holograms.

## 5. Conclusions

Photochromic materials give interesting opportunities as substrates for the manufacturing of rewritable Computer-Generated Holograms (CGHs). Indeed, phase and amplitude holograms are demonstrated and binary and grayscale pattern can be easily transferred. In order to make high quality holograms, the optimization of both the photochromic material and the writing procedure is necessary. As for the material optimization, the combination of a kinetic model and experimental UV-vis data makes possible the development of a computational tool to predict the performances in terms of transparency contrast of photochromic films. In this way, a balanced choice of the film thickness and photochromic content leads to high efficiency amplitude holograms. In addition, the versatility in the synthesis of photochromic diarylethenes provides many possibilities in tuning the spectral position and intensity of the band along the whole visible region. As for the phase hologram and the modulation of the refractive index, important guidelines are provided in order to maximize the efficiency. We also highlighted that another crucial aspect is the writing strategy; here both a reconfigurable mask approach based on a DMD chip and a direct laser writing machine are reported. The former is more suitable for the realization of grayscale patterns, but suffers from a low spatial resolution; the latter is more suitable for binary patterns and provides a much larger spatial resolution in the case of large area CGHs.

## Figures and Tables

**Table 1:** Absorption maxima (λ) and molar extinction coefficient (ε) of diaryletenes shown in Figure 5 in their two isomeric forms (uncolored and colored). Absorption spectra measured in hexane solution (* in EtOH).

Compound	Uncolored Form		Colored Form	
	λ_UV_ (nm)	ε_UV_ (M^−1^ cm^−1^)	λ_VIS_ (nm)	ε_VIS_ (M^−1^ cm^−1^)
**1**	278	34,660	575	14,990
2 *	288	38,720	584	16,400
**3**	328	40,450	608	14,450
**4**	293	43,610	582	20,050
**5**	297	41,660	583	21,110
**6**	294	36,580	582	16,940
**7**	296	35,980	592	18,010
**8**	320	49,960	607	24,300
**9**	353	51,040	613	28,100
**10**	315	27,670	598	9790
**11**	315	33,060	591	11,425
**12**	330	44,230	642	25,860
**13**	305	37,970	597	18,070
**14**	305	35,390	602	16,620
**15**	251	21,410	546	10,910
**16**	282	30,780	587	15,420
**17**	310	28,190	592	16,750
**18**	318	32,480	598	21,490
19 *	320	32,420	608	19,090
**20**	301	30,640	519	10,810
**21**	316	23,700	534	11,800
**22**	329	37,310	549	19,400
23 *	304	35,050	530	12,690
24 *	330	31,520	553	15,820
